# Mammary adipose dysfunction in the dual epidemic of obesity and breast cancer

**DOI:** 10.1093/carcin/bgaf053

**Published:** 2025-09-03

**Authors:** Elvina Jeyakumar, Sathyavathi Sundararaju, Stephanie Annett, Mohamed A Elrayess

**Affiliations:** College of Medicine, QU Health, Qatar University, Doha, PO Box 2713, Qatar; School of Pharmacy and Biomolecular Sciences, Royal College of Surgeons in Ireland, 123 St Stephen’s Green, Dublin D02 YN77, Ireland; College of Medicine, QU Health, Qatar University, Doha, PO Box 2713, Qatar; School of Pharmacy and Biomolecular Sciences, Royal College of Surgeons in Ireland, 123 St Stephen’s Green, Dublin D02 YN77, Ireland; College of Medicine, QU Health, Qatar University, Doha, PO Box 2713, Qatar; Biomedical Research Center, QU Health, Qatar University, Doha, PO Box 2713, Qatar

**Keywords:** mammary adipose tissue, breast cancer, obesity, adipokine imbalance, metabolic dysfunction, menopause

## Abstract

Breast cancer (BC) is one of the leading causes of death among women, with obesity being a significant factor. Mammary adipose tissue (MAT) dysfunction in obesity creates a tumor-supportive environment, leading to increased risk. In obesity, MAT undergoes significant changes, including increased adiposity, chronic inflammation, aromatase overexpression, insulin resistance, and altered adipokine signaling, collectively fostering a protumorigenic microenvironment. The interaction between adipocytes and cancer cells further exacerbates BC progression through metabolic crosstalk and immune evasion. This review examines the role of MAT dysfunction in BC incidence and progression, in obesity. Interestingly, obesity appears to have a paradoxical effect on BC risk, offering a potentially protective role in premenopausal women, but increased risk in postmenopausal women, primarily due to differences in estrogen levels. Addressing the metabolic, inflammatory, and hormonal abnormalities in obese MAT can aid in enabling the development of precision therapies that reduce BC risk and improve treatment outcomes in obese patients.

## Graphical Abstract

**Graphical Abstract:**
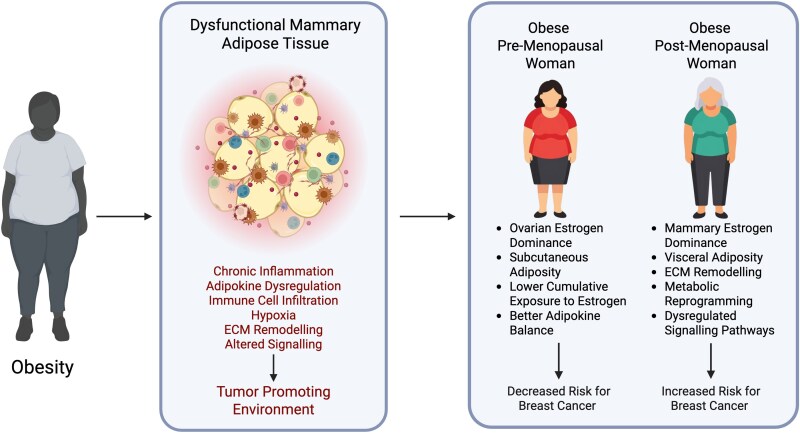

## 1. Introduction

Breast cancer (BC) is the most prevalent carcinoma among women worldwide, with nearly 2.3 million new cases reported in 2022, and a leading cause of cancer-related mortality, representing a significant public health concern [1]. Obesity is a major risk factor, contributing to 4%–8% of all cancer cases [2]. It is characterized by systemic metabolic dysfunction, marked by insulin resistance (IR) and inflammation, creating a protumorigenic environment, ultimately increasing morbidity and mortality rates in cancer patients. The rising rates of both BC and obesity highlight the need to address this dual epidemic, as BC incidence is directly linked to obesity prevalence [3, 4].

In healthy women, mammary adipose tissue (MAT) regulates breast tissue homeostasis by secreting hormones and adipokines [5, 6]. In obesity, MAT changes—such as hypertrophy, hypoxia, and increased aromatase activity—boost estrogen, inflammation, and adipokine release, fostering a tumor-promoting environment. Dysfunctional MAT links obesity to BC, acting as both a risk factor and a prognostic marker [3, 5, 6].

Recently, it has been observed that MAT dysfunction’s impact on BC risk varies by menopausal status. In premenopausal women, ovarian estrogen predominates, and obesity-related factors like hyperinsulinemia and inflammation have a greater effect. In postmenopausal women, estrogen production shifts to adipose tissue, raising aromatase expression and local estrogen levels in MAT, worsening dysfunction and heightening BC risk [7–9].

Epidemiological studies consistently show that obesity increases postmenopausal BC and worsens outcomes at any age. This association is driven by systemic metabolic alterations, chronic inflammation, and hormonal dysregulation, which are inherent to obesity, and is correlated with an increased risk of recurrence and a worse prognosis of cancer [7, 10–13]. Postmenopausal obese women have ∼30% higher BC risk compared with nonobese women, and those with a body mass index (BMI) of over 35 have 1.58 times the risk of developing invasive BC compared with women of normal weight [10, 14].

In contrast, a study by Schoemaker *et al*. (2018) involving over 125 000 premenopausal women found that higher BMI and adiposity were associated with lower BC risk, particularly in hormone receptor–positive cases. These differing risk profiles underscore the importance of menopausal status in obesity-related BC risk [15]. Beyond BMI, metabolic factors like leptin levels have been linked to BC risk. A meta-analysis by Pan *et al*. [16] examined 35 case–control and cohort studies and confirmed a strong association between higher leptin levels and increased BC risk, particularly in overweight and obese individuals, although findings in premenopausal women were inconsistent.

Emerging evidence suggests that obesity may protect against BC in premenopausal women but significantly increases risk in postmenopausal women, especially for estrogen receptor–positive (ER+) tumors [12, 17]. This highlights the need for an understanding of how the menopausal state affects MAT-driven mechanisms in BC progression. This review aims to synthesize the current knowledge on how obesity-driven MAT dysfunction contributes to BC development and progression, particularly differences by menopausal status, and to elucidate the pathways implicated in the process. Recognizing the obesity-associated changes in the MAT will provide insights into BC etiology and help identify potential targets for prevention and therapeutic intervention in this population.

### 1.1. Mammary adipose tissues role in breast health

MAT is a specialized adipose tissue that acts as an energy reserve that provides structural support to the tissue and secretes hormones, adipokines, and growth factors that influence the growth and differentiation, angiogenesis, and tissue remodeling of the mammary gland [18]. Key aspects of its endocrine activity include the following:

1. Adipokine production: The MAT produces adipokines like leptin and adiponectin, which exert opposing effects on breast epithelial cells. Leptin is a proinflammatory and protumorigenic adipokine that promotes cell proliferation, migration, and angiogenesis, whereas adiponectin is an anti-inflammatory adipokine with protective effects, inhibiting cell proliferation, promoting apoptosis, and enhancing insulin sensitivity of the tissue [18, 19].
2. Estrogen synthesis: The adipocytes in the mammary tissue secrete an enzyme—aromatase, which is responsible for the conversion of androgens to estrogens. This stimulates an increase in estrogen production in the breast tissue. Usually, aromatase expression in MAT is a tightly regulated process. This ensures that the physiological functions of MAT are supported, but excessive estrogen production, which could lead to uncontrolled cell growth, is prevented [20, 21].
3. Crosstalk with breast epithelial cells: MAT-derived factors like insulin-like growth factor-1 (IGF-1) and vascular endothelial growth factor (VEGF) directly influence the behavior of breast epithelial cells by activating signaling pathways such as Janus kinase/signal transducers and activators of transcription (JAK/STAT), phosphoinositide 3-kinase/protein kinase B (PI3K/AKT), and mitogen-activated protein kinase (MAPK), which regulate proliferation and survival [19].

MAT actively interacts with the immune cells such as macrophages, T cells, and natural killer cells to produce cytokines to modulate inflammation and immune responses. These regulatory functions of MAT are critical due to the proximity of MAT to mammary epithelial cells, where subtle perturbations in signaling influence tissue health and disease [22].

MAT plays a crucial role in breast development and function by providing essential growth factors and hormones, driving the growth of mammary glands. However, obesity significantly alters MAT’s functions—through adipocyte hypertrophy, macrophage infiltration, and increased aromatase activity, potentially increasing the risk of BC. These alterations make MAT a crucial component that can directly influence BC risk.

## 2. Obesity, mammary adipose tissue dysfunction, and breast cancer

### 2.1. Obesity

Obesity, the excessive accumulation of body fat—adipose tissue, is a well-established risk factor for cancer, with its influence extending beyond the effects of adipose tissue accumulation. A significant increase in adipose tissue mass can result in a variety of metabolic and hormonal changes that create an environment conducive to BC development [2].

The relationship between obesity and BC has been the subject of extensive research over past decades. While multiple factors contribute, the influence of BMI on cancer risk is significant, with numerous studies demonstrating that a higher BMI correlates with an increased incidence of BC [6, 10, 23]. Extensive research links obesity to an elevated risk of BC through three interconnected mechanisms: hyperinsulinemia, systemic metabolic dysfunction, and chronic low-grade inflammation. The dynamic interaction between these three important pathological processes creates a pro-oncogenic environment that fuels tumor initiation and progression [24, 25].

#### 2.1.1. Hyperinsulinemia and phosphoinositide 3-kinase/mitogen-activated protein kinase pathways

IR, a key factor of obesity-associated metabolic dysfunction, significantly impacts BC progression. Insulin has antiapoptotic characteristics that promote tumor-invasive activity. In addition, BC cells were observed to overexpress insulin receptors 6- to 10-fold compared with normal cells. Insulin, by activation of its receptors, triggers the PI3K and MAPK pathways, promoting cell growth and survival [26]. In obesity, compensatory hyperinsulinemia amplifies these effects. Dysregulation of these pathways under IR conditions leads to chronic insulin receptor activation, increased glucose uptake, proliferation, reduced apoptosis, and tumorigenesis. Hyperactivation of MAPK further drives uncontrolled growth, angiogenesis, and immune evasion, accelerating cancer progression [11, 27, 28].

Hyperinsulinemia increases IGF-1 bioavailability, especially in obese individuals. IGF-1 enhances tumor cell proliferation and survival, while inhibiting apoptosis, promoting BC progression and metastasis. Elevated IGF-1 levels also exacerbate IR [28, 29]. Additionally, hyperinsulinemia reduces sex hormone-–binding globulin (SHBG) and thereby increases circulating estrogen, which promotes proliferation, survival, and invasion, especially in ER+ BC [30, 31]. Together, hyperinsulinemia and IGF-1 elevation create a tumor-supportive environment by stimulating oncogenic signaling, angiogenesis, and immune evasion. This underscores the role of IR in obesity as a key driver of BC progression.

#### 2.1.2. Chronic low-grade inflammation driving breast cancer progression

Chronic low-grade inflammation, a hallmark of obesity, drives BC initiation and progression. In obese individuals, enlarged adipocytes undergo necrotic-like death, triggering macrophage infiltration and formation of crown-like structures (CLSs). These CLSs release proinflammatory cytokines (TNF-α, IL-6, IL-1β) that activate nuclear factor-kappa B (NF-κB) and STAT3 pathways, promoting tumor initiation, angiogenesis, and metastasis. This inflammatory environment induces DNA damage, epigenetic changes, and oxidative stress, increasing cancer risk through reactive oxygen species (ROS)-mediated oncogenesis [32–34].

Chronic inflammation in the MAT promotes proliferation and survival of cancer cells and disrupts immune surveillance, allowing cancer cells to evade detection [7, 32]. In obesity, chronic inflammation causes immune exhaustion, affecting T-cell function and natural killer cell maturation. It also recruits immunosuppressive cells such as myeloid-derived suppressor cells, regulatory T-cells, and tumor-associated macrophages that can suppress antitumor responses [35, 36]. Because of elevated baseline levels of inflammatory mediators, obese individuals exacerbate these effects, which results in an inflamed, immune-suppressed environment favoring tumor growth. Increased macrophage infiltration and proinflammatory signaling enhance cancer cell survival and metastasis, making obesity a significant risk factor for BC development, with poor outcomes.

#### 2.1.3. Systemic metabolic dysfunction

Adipose tissue secretes a bioactive molecule, called adipokines, which regulate metabolism, inflammation, and cellular homeostasis. In obesity, the levels of protumorigenic adipokines like leptin increase, while protective ones like adiponectin decrease. Overexpression of leptin activates oncogenic pathways (JAK/STAT, PI3K/AKT, MAPK), enhancing BC progression, whereas adiponectin downregulation negates its anti-inflammatory and antiproliferative effects, leaving the MAT vulnerable to malignancy [3, 37, 38].

### 2.2. Mammary adipose tissue dysfunction in obesity

In obese conditions, MAT undergoes structural and functional changes, including increased adiposity, chronic inflammation, hypoxia, estrogen overexpression, adipokine imbalance, IR, and elevated IGF-1 levels, creating a protumorigenic environment that drives BC progression [5, 39]. This section explores the key changes occurring in MAT due to obesity and the tumor-promoting mechanisms associated with these alterations, resulting in dysfunctional MAT.

Excessive adipose expansion leads to adipocyte hypertrophy and local hypoxia, stabilizing hypoxia-inducible factors and increasing angiogenic and inflammatory mediators like VEGF, IL-6, and TNF-α. These factors promote angiogenesis, extracellular matrix (ECM) remodeling, and chronic inflammation. Hypoxia amplifies inflammation by releasing cytokines and chemokines that recruit immune cells to the tumor microenvironment (TME). A hallmark of this state is the formation of CLSs, which sustain inflammation, recruit proinflammatory immune cells, promote genetic instability, enhance proliferation, and inhibit apoptosis, ultimately driving BC progression [5, 32, 39, 40].

In obese MAT, increased aromatase expression increases CLS, driven by IL-6, a proinflammatory cytokine, leptin, and hypoxia. This creates a self-amplifying loop where cytokines induce aromatase expression, increasing estrogen levels, fueling cancer cell proliferation, and further cytokine release. Leptin amplifies aromatase activity through STAT3 activation, increasing the local bioavailability of estrogen. The resulting excess inflammation and estrogen-rich environment, exacerbated in obesity, promotes cell cycle progression, inhibits apoptosis, and drives angiogenesis, particularly in ER+ cancers. Estrogen also suppresses antitumor immunity and upregulates VEGF. The decreased adiponectin-to-leptin ratio in obesity further skews the breast microenvironment toward a protumorigenic state. It interferes with the regulation of breast epithelial cells, facilitating the onset and progression of cancer [3, 22, 41].

**Figure 1:**
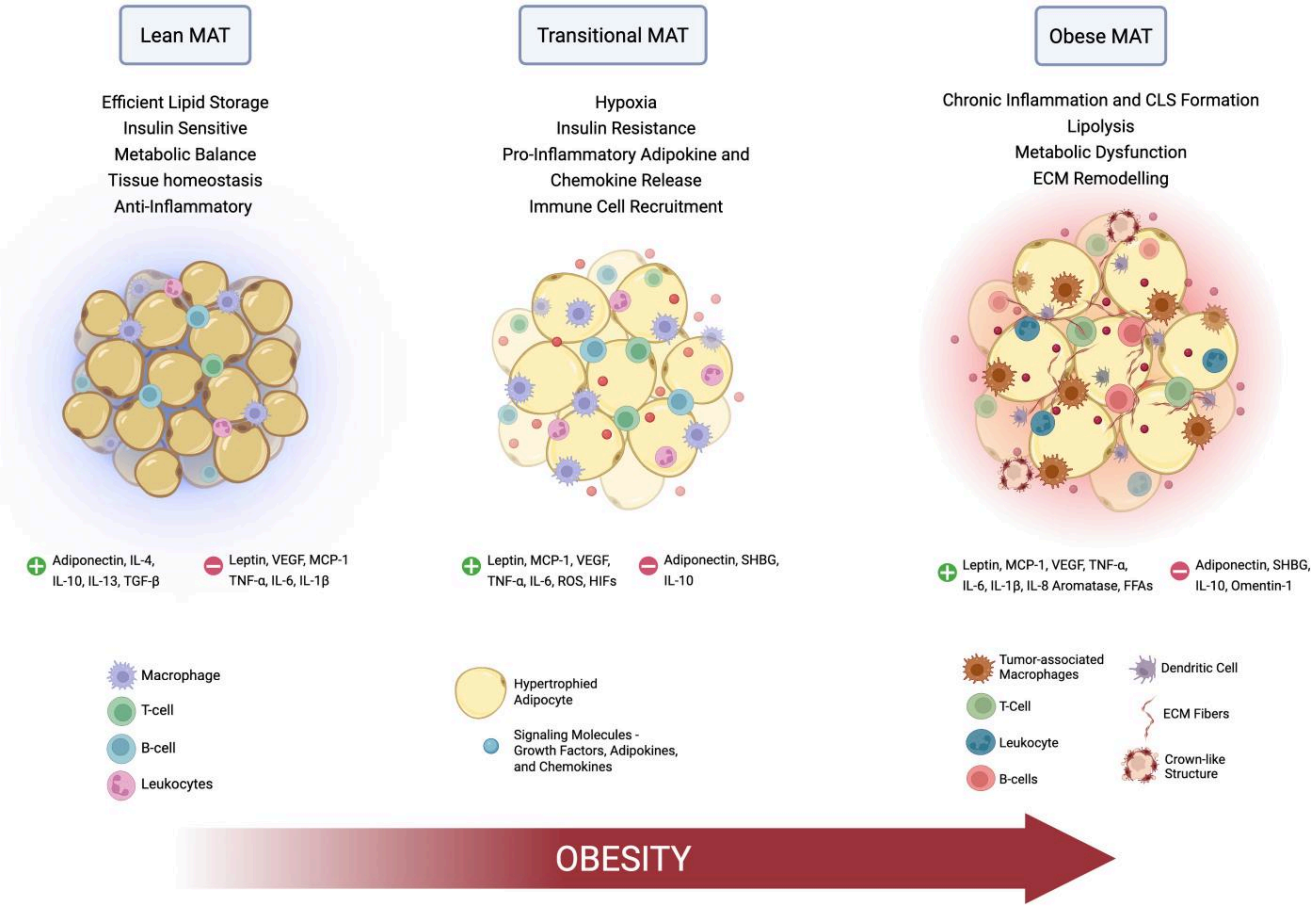
Schematic representation of MAT remodeling in obesity. Lean MAT, characterized by efficient lipid storage, insulin sensitivity, and metabolic balance, ensuring homeostasis of the MAT, which is further supported by anti-inflammatory adipokines (adiponectin, IL-4, IL-10, IL-13, and TGF-β) and immune cells (macrophages, leukocytes, T and B cells). As the cells begin to hypertrophy under overweight conditions, the MAT enters a transitional state, exhibiting early signs of dysfunction, like hypoxia [marked by the release of hypoxia-inducible factors (HIFs)], IR, release of various signaling molecules like VEGF, and proinflammatory adipokines [leptin, VEGF, monocyte chemoattractant-1 (MCP-1), TNF-α and IL-6], and ROS, accompanied by a reduced secretion of protective factors like adiponectin, SHBG, and IL-10. With progression to obesity, the MAT undergoes significant remodeling. Obese MAT is characterized by chronic inflammation and CLS formation, lipolysis, metabolic dysfunction, and ECM remodeling. This is accompanied by a marked increase of inflammatory mediators (leptin, MCP-1, VEGF, IL-6, IL-1β, and IL-8) and a shift in immune cell populations, now including tumor-associated macrophages and dendritic cells. Collectively, these changes create an environment favorable to cancer development and progression (Image created using BioRender.com)

Collectively, these processes disrupt normal signaling pathways and thereby tissue homeostasis, resulting in the creation of an environment conducive to cancer development and progression. The key aspects of this transition, from a lean to an obese dysfunctional state, are summarized in Fig. 1. Understanding these pathways supports targeted interventions aimed at disrupting the tumor-supportive effects of MAT dysfunction.

However, MAT dysfunction is not alone in this. Recent evidence suggests that the interactions between the cancer cells and the surrounding adipocytes in the mammary gland play a critical role in modulating the TME and encouraging tumor growth.

## 3. Adipocyte-cancer cell crosstalk

The interaction between cancer cells and surrounding adipocytes is crucial for TME remodeling in dysfunctional MAT in cancer. The dynamic, bidirectional exchange of signals results in the formation of cancer-associated adipocytes (CAAs) that promote tumor growth, invasion, and metastasis. The communication between adipocytes and cancer cells involves metabolic, inflammatory, and structural alterations in both cell types, resulting in a protumorigenic niche [38].

The formation of CAAs starts with the BC cells inducing metabolic reprogramming in nearby adipocytes. In response to the interaction with the cancer cells, adipocytes increase lipolysis, in turn, increasing the levels of free fatty acids (FFAs) released into the TME. These FFAs are the primary source of energy for the cancer cells, which primarily use fatty acid oxidation to support their growth and proliferation. Cancer cells also induce adipocytes to release glucose and lactate as additional energy sources. This reprogramming alters lipid-laden adipocyte morphology, disrupts energy homeostasis, and interferes with endocrine functions, resulting in decreased beneficial adipokines, while increasing proinflammatory cytokines. The reduced lipid content locally contributes to ECM remodeling, facilitating cancer cell invasion and migration. In obese individuals, this process is amplified, due to the increased availability of FFAs from hypertrophic adipocytes. The excess FFAs enhance cancer cell metabolism, promoting their proliferation and survival even in nutrient-deprived conditions. These metabolic alterations induced by the cancer cells facilitate the transformation of adipocytes into CAAs [38, 42].

**Figure 2:**
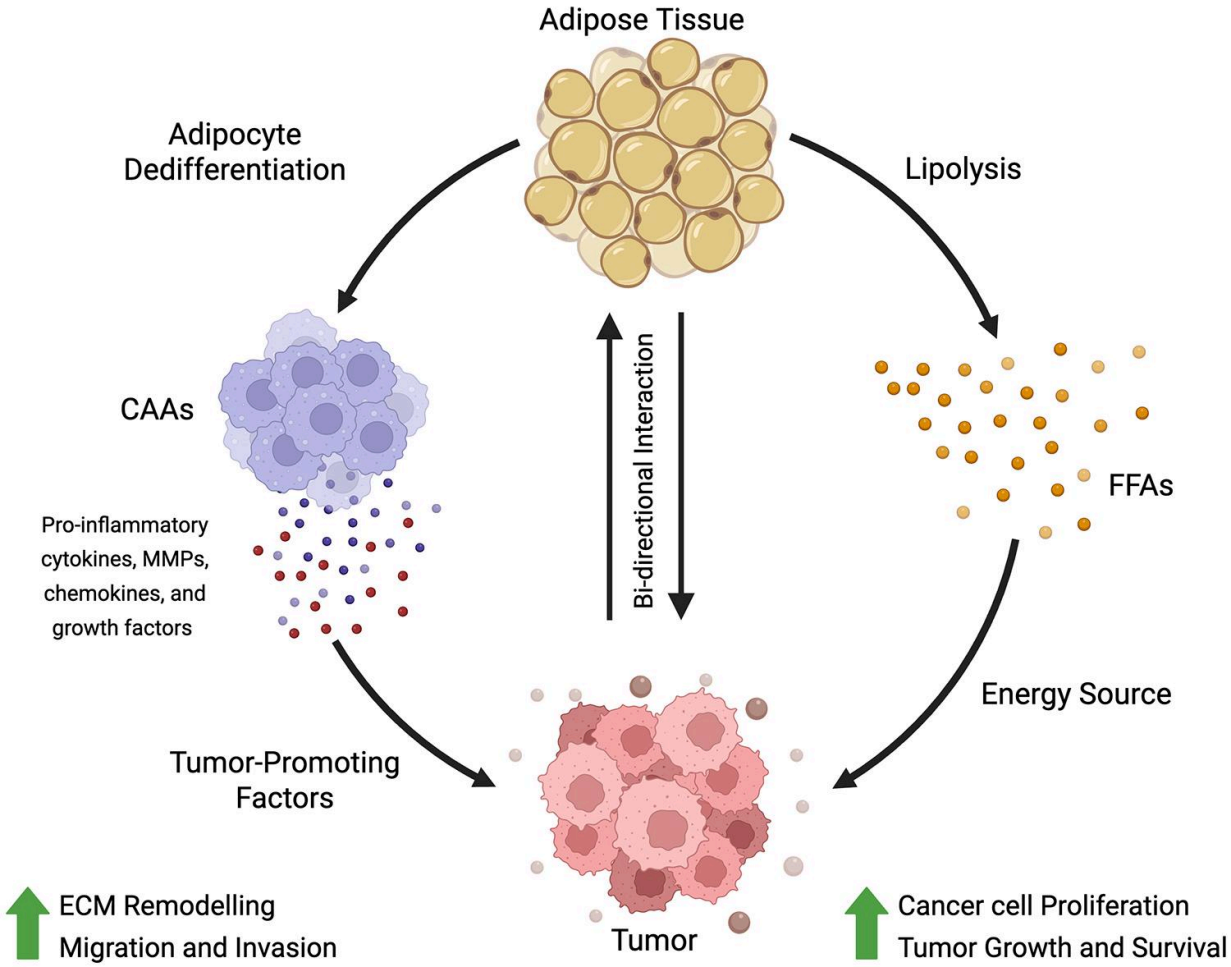
Bi-directional interaction between cancer cells and adipocytes. The interaction between cancer cells and adipocytes is bidirectional, forming a vicious cycle that creates a positive feedback loop supporting tumor development. Initially, cancer cells increase lipolysis in adipocytes, releasing FFAs that serve as an energy source, to support and enhance cancer cell growth and survival, especially in nutrient-poor environments. Consequently, adipocytes are reprogrammed into CAAs, adopting a protumorigenic phenotype. CAAs produce inflammatory cytokines, chemokines, and growth factors that support cancer progression. They also secrete MMPs that contribute to ECM remodeling, inducing structural changes that facilitate cancer cell invasion. This bidirectional interaction ensures that cancer cells not only sustain their growth but also reshape the tumor microenvironment to their advantage, making the disease more aggressive [4, 38]. (Image created using BioRender.com)

Following the metabolic alteration to trigger CAA formation, the adipocytes undergo dedifferentiation of the, induced by the cancer cells, through the adipocyte-mesenchymal transition. During this transition, driven by the YAP/TAZ signaling pathway, the mature adipocytes lose their defining characteristics like lipid storage capacity and expression of differentiation markers like PPAR-γ and C/EBP-α, and instead acquire a fibroblast-like phenotype, becoming smaller and irregular in shape, with reduced lipid storage. These dedifferentiated adipocytes secrete cytokines, chemokines, and growth factors that are crucial in sustaining cancer cell proliferation and invasion. Along with the phenotypic transformation, they also undergo cytoskeletal remodeling that promotes the invasive and migratory features of the cells and enhances their ability to interact with cancer cells. CAAs can also induce epithelial-to-mesenchymal transition in the tumor cells, significantly increasing their metastatic potential. Additionally, the new CAAs secrete inflammatory cytokines, chemokines, and growth factors that support cancer progression [42–44]. This bi-directional communication between the cancer cells and adipocytes is depicted in Fig. 2.

In obese individuals, due to increased availability of FFAs from hypertrophic adipocytes, cancer cell metabolism is enhanced, promoting proliferation and survival even in nutrient-deprived conditions, highlighting how cancer cells exploit their microenvironment for survival and progression [38, 42, 45].

Another key pathway is leukemia inhibitory factor (LIF)-chemokine (C-X-C motif) ligand (CXCL) pathway, which forms a positive feedback loop for adipocyte–cancer cell interaction. CAAs release the LIF, promoting BC cell migration and invasion through the STAT3 signaling pathway. Activated STAT3 induces CXCL production in adipocytes, which triggers the MAPK/NF-κB/STAT3 cascade, enhancing LIF expression and creating a self-reinforcing cycle that drives BC tumor progression [46–48].

CAAs intrinsically secrete proinflammatory cytokines, chemokines, and growth factors (Table 1) via a tumor-promoting feedback loop, which promote tumor growth, immune evasion, tissue remodeling, and sustained cancer proliferation. In obesity, increased secretion of these mediators exacerbates adipose tissue dysfunction, amplifying inflammation, immune cell recruitment, and remodeling to create a tumor-supportive environment conducive to growth, invasion, and metastasis [38, 43, 55].

**Table 1:** Key molecular mediators involved in the tumor-promoting feedback loop within the TME.

Category	Key factors	Function in tumor progression	References
Proinflammatory cytokines	IL-6, IL-1β, TNF-α	Sustain chronic inflammation within the tumor microenvironment, promoting cancer cell survival and proliferation.	[24, 49, 50]
Chemokines	CCL2, CCL5	Recruit immune cells (e.g. macrophages) to the tumor site, contributing to immune evasion and tumor progression.	[24, 45]
Growth factors	VEGF EGF, IGF-1	Stimulate angiogenesis, increasing blood supply and nutrient availability to the tumor.	[49, 50]
Adipokines	Leptin, adiponectin	Stimulate angiogenesis, promote chronic inflammation, increase cell proliferation, and enhance tumor progression by activating oncogenic pathways.	[51, 52]
Hypoxia-inducible factors (HIF)	HIF-1α, HIF-2α	Activate genes that help tumor cells survive and thrive in low-oxygen conditions, promote angiogenesis, enhance glucose metabolism, and increase tumor cell invasion and metastasis abilities.	[53, 54]

In addition, CAAs secrete matrix metalloproteinases that degrade existing ECM, enabling cancer cell invasion and metastasis. They also promote fibrotic tissue deposition, creating a protective scaffold for cancer cell proliferation and increasing tumor stiffness. These changes facilitate tumor progression and contribute to treatment resistance [56–58].

Collectively, the bidirectional interaction between the nearby adipocytes and the BC cells transforms MAT into a highly tumor-supportive niche. By driving chronic inflammation, immune suppression, and ECM remodeling, the CAAs accelerate tumor progression. These findings suggest the potential of targeting the implicated signaling pathways like the leptin-PI3K/AKT and LIF-CXCL, or blocking matrix metalloprotease (MMP) activity, for novel therapeutic options in obesity-associated BC.

## 4. Immune dysregulation in mammary adipose tissue and breast cancer progression

Beyond the metabolic and phenotypic alterations that support cancer cells, CAAs contribute to a cancer-promoting TME by sustaining inflammation and interacting with surrounding cells, especially immune cells, to promote immune evasion, tumor survival, and progression. Infiltrating immune cells—such as macrophages, T cells, and neutrophils—maintain this inflammatory state, supporting tumor growth. This immune dysregulation, alongside adipocyte dysfunction, is central to shaping a protumorigenic TME. Table 2 summarizes the impact of obesity on a few key immune cells, highlighting the pathological changes that drive BC progression in obese conditions.

**Table 2:** Immune cell function in healthy versus obese conditions.

Cell type	Healthy conditions	Obese conditions	Pathological changes in obese conditions promoting breast cancer	References
Tumor-associated macrophages (TAMs)	TAMs play a crucial role in clearing dead cells and maintaining tissue homeostasis.	TAMs infiltrate MAT and form CLS around necrotic adipocytes, releasing proinflammatory cytokines that sustain chronic inflammation within the tumor microenvironment.	The chronic inflammatory state reprograms TAMs into a protumorigenic phenotype, promoting angiogenesis, immune evasion, ECM remodeling, and epithelial-to-mesenchymal transition, facilitating metastasis. TAMs also suppress cytotoxic T-cell responses, impairing the immune system’s ability to target and destroy cancer cells. In addition, TAMs promote the emergence of cancer stem cells, which are known for their ability to form tumors and metastasize.	[3, 59]
T-cells	In healthy conditions, regulatory T-cells maintain immune tolerance and prevent excessive immune responses. CD8+ cytotoxic T-cells help eliminate abnormal or cancerous cells.	Obesity is associated with an increased accumulation of regulatory T-cells in MAT, leading to the suppression of antitumor immune responses. CD8+ cytotoxic T-cells become dysfunctional, further impairing immune surveillance.	The suppression of immune responses allows for impaired immune clearance, allowing tumor immune evasion and promoting cancer progression.	[60, 61]
Neutrophils	Neutrophils function as a key component of the innate immune system, destroying pathogens via phagocytosis, formation of neutrophil extracellular traps (NETs), and the release of ROS and cytokines.	Increased neutrophil infiltration in MAT, exacerbating local inflammation.	Increased ROS secretion induces oxidative stress and DNA damage, driving tumor initiation. Proinflammatory cytokines recruit immune cells, amplifying inflammation and angiogenesis. In dysfunctional adipose tissue, NETs, instead of trapping cancer cells, facilitate their migration and invasion, promoting metastasis.	[59, 62, 63]
Dendritic cells (DCs)	The DCs function as antigen-presenting cells that regulate immune responses and maintain immune homeostasis.	Chronic inflammation in obesity alters DC function, shifting them toward a proinflammatory phenotype. This leads to increased production of cytokines such as IL-6, TNF-α, and IL-12, which promote tumorigenesis. Obesity also increases ROS production by DCs.	Dysfunctional DCs have impaired antitumor immunity, including reduced antigen presentation and T-cell activation, promoting a proinflammatory environment, increasing oxidative stress, and altering immune infiltration within the adipose tissue. Dysfunctional DCs can also transform TAMs into a protumor state, further suppressing antitumor responses and allowing cancer cells to evade immune surveillance.	[64, 65]

In summary, the changes occurring because of dysfunctional MAT significantly alter the immune landscape in the TME, shifting the balance toward chronic inflammation and immune suppression. This dysregulation of the immune system, characterized by an increased infiltration of immune cells, secretion of proinflammatory cytokines, impaired function and exhaustion of immune cells, and a suppressed antitumor immune response, creates a microenvironment that is highly supportive of BC development and progression, as well as poor prognosis [64]. These immune alterations, driven by obesity, further strengthen the obesity-BC link, emphasizing the critical role of immune dysregulation in tumor development.

Beyond the established link between obesity-induced MAT dysfunction, altered immune landscape, and BC progression, hormonal factors, particularly menopausal status, introduce another layer of complexity to this intricate relationship and can influence the TME and BC progression.

## 5. Mammary adipose tissue dysfunction in premenopausal obese women

While obesity is a well-established risk factor for BC, in premenopausal women, it has a complex risk profile. The dysfunction in MAT—characterized by inflammation, hormonal imbalance, and metabolic alterations—is counteracted by protective mechanisms inherent to the premenopausal hormonal environment.

### 5.1. Protective effects of obesity on breast cancer risk premenopause

The largest multicenter study was carried out by The Premenopausal Breast Cancer Collaborative Group, exploring the association between obesity and BC risk in premenopausal women. The study examined data from 758 592 premenopausal women with an average follow-up period of 9.3 years and identified a 4.2-fold elevated risk of BC in underweight women relative to obese women under 24 years of age, and a 1.9- to 2.5-fold increased risk in older premenopausal women. Additionally, there was an estimated 12%–23% decrease in premenopausal BC risk for each 5 kg/m^2^ increase in BMI, indicating an inverse correlation between obesity and BC risk in younger women [15]. Multiple other studies [66, 67], including a cohort study of 125 188 premenopausal Korean women, support the finding that increased BMI is associated with a reduced risk of developing BC [17]. Another meta-analysis of over 2 million women reported an 8% reduction in BC risk per 5 kg/m^2^ BMI increase in premenopausal women, contrasting with a 12% increase in postmenopausal women [68]. Studies also report that lower mammographic density in obese premenopausal women may contribute to reduced risk, as higher density is a strong risk factor for BC [11].

This inverse relationship between obesity and BC can be attributed to the hormonal and metabolic changes specific to premenopausal women. In premenopausal women, estrogen is primarily produced by the ovaries. Obesity can lead to estrogen accumulation in the enlarged fat cells, lowering circulating bioavailable estrogen, thereby potentially reducing BC risk. Generally, premenopausal women also have a more favorable adipokine profile, with relatively high adiponectin levels that help neutralize the cancer-promoting effects of leptin. Estrogen also supports insulin sensitivity, encouraging glucose uptake and lowering inflammation. Menstrual cycle fluctuations and elevated adiponectin contribute to better glucose control, decreasing the likelihood of hyperinsulinemia and its tumor-promoting effects. Increased levels of SHBG in premenopausal women, which binds to estrogen and reduces the active hormone in circulation, counteract the impact of locally produced estrogen via aromatase in MAT, especially in obesity. Additionally, obesity in these women is often linked to anovulatory cycles and decreased progesterone production, thereby reducing cumulative exposure of MAT cells to estrogen. These factors collectively counteract the tumor-promoting effects of locally produced estrogen in MAT, contributing to a lower BC risk in obese premenopausal women [8, 11, 69].

### 5.2. Risks associated with mammary adipose tissue dysfunction in premenopausal women

Although a protective effect of MAT dysfunction in BC in obese premenopausal women has been observed and reported, there still are other risks associated with MAT dysfunction in this population. The metabolic dysregulation in obesity enhances protumorigenic signaling and BC risk even in premenopausal women, through estrogen-independent mechanisms such as IR, chronic inflammation, and immune suppression.

As discussed previously, dysfunctional MAT fosters a protumorigenic microenvironment by promoting chronic inflammation and immunosuppression, driven by CLS formation, altered immune cell function, and proinflammatory cytokine release. It is exacerbated by hypoxia in hypertrophic adipocytes and adipokine imbalance–driven activation of protumorigenic pathways. In addition, obesity-induced ECM changes, metabolic reprogramming of CAAs, and adipocytes create an environment conducive for cancer cell growth. Emerging research also suggests that obesity can alter the breast tissue microbiome, which, in turn, can influence MAT function, immune responses, and potentially increase BC risk [70].

Obesity among premenopausal women has also been associated with an increased risk of more aggressive BC subtypes, such as triple-negative BC or hormone receptor–negative BC, due to the lack of hormone receptors. These cancers are more likely to develop as larger tumors with higher cell proliferation rates, lymph node metastasis, and vascular infiltration—resulting in poor prognosis [71, 72].

In essence, obesity exerts a dual influence on cancer risk in premenopausal women. The hormonal environment characterized by dominant ovarian estrogen, reduced progesterone, and beneficial adipokines, can counteract the tumor-promoting effects of MAT dysfunction in hormone receptor–positive BCs. However, the risk remains for more aggressive subtypes like triple-negative BC. Moreover, ECM remodeling, metabolic alterations, and epigenetic modifications can amplify the impact of MAT dysfunction on BC progression [21, 40]. This complexity underscores the need for tailored interventions considering menopausal status. Understanding this protective versus risk-enhancing effects of MAT dysfunction will guide the development of prevention strategies and inform personalized monitoring approaches in these patients. The next section will delve into how this risk profile changes post menopause when ovarian estrogen production ceases and MAT dysfunction becomes a more significant driver of BC progression.

## 6. Mammary adipose tissue dysfunction in postmenopausal obese women

Post menopause, estrogen production declines and shifts from the ovaries to the adipose tissue. In obese women, this transition is amplified by heightened aromatase activity in the MAT, resulting in increased local estrogen exposure. Coupled with obesity-driven inflammation, metabolic reprogramming, and adipokine imbalance, this creates a protumorigenic environment that significantly increases BC risk.

### 6.1. Increased risk of breast cancer development in postmenopausal women

MAT dysfunction in postmenopausal obese women plays a significant role in increasing BC risk, particularly for hormone receptor–positive tumors. As ovarian function declines, estrogen synthesis shifts to MAT, where obesity-linked aromatase activity increases local estrogen. Consequently, postmenopausal obese women face a higher risk of BC due to increased estrogen production from the MAT, a key driver of tumor initiation and progression. This association is supported by multiple large-scale studies and meta-analyses. The Million Women Study, which followed 1.2 million UK women aged 50–64 years, found that obese postmenopausal women had an ∼30% higher risk of BC compared with nonobese women [73]. Other studies reported that women with a BMI >35 had 1.58 times the risk of developing invasive BC, and an even stronger association with hormone receptor–positive cancers (1.86 times higher risk) compared with women of normal BMI [10, 13].

Obese postmenopausal women have a higher proportion of adipocytes in their breasts. When estrogen, particularly estradiol, is sequestered within these adipocytes, it can trigger a differential expression of ER expression locally. Obesity also creates a tumor-promoting environment via increased CLS formation, proinflammatory cytokines, adipokine imbalance, and hyperinsulinemia through insulin/IGF-1 pathways, enhancing tumor growth, angiogenesis, immune suppression, cell proliferation, and estrogen bioavailability by suppressing SHBG. Age-associated decrease in insulin sensitivity further worsens these effects [31]. In addition, menopause promotes fat redistribution with greater accumulation in the visceral area. This increase in visceral fat, as opposed to subcutaneous fat accumulation in premenopausal women, is associated with a less favorable adipokine profile, dyslipidemia, and IR.

Obesity also disrupts normal cell signaling and gene expression within the MAT, which drives remodeling of the ECM to favor tumor initiation and progression. Stromal cells within the MAT, which primarily support the growth of normal breast epithelial cells, can also support the metastatic activity of the BC cells, when they release growth factors in response to the local hormone levels—including increased estrogen [74]. The combination of menopause and obesity also leads to changes in the ECM. Aged breast tissue shows reduced collagen XV and V, increased stiffness, and thicker, more curved collagen fibers. There is also increased fibrosis of the tissue from the chronic inflammation. These changes in the ECM can drive normal mammary epithelial cells toward a more invasive and cancer-like phenotype [75]. Metabolic reprogramming in obesity leads to increased lipolysis and altered glucose metabolism, providing essential nutrients for tumor development and growth. Epigenetic changes in dysfunctional MAT can also affect gene expression patterns related to cell proliferation, apoptosis, and immune responses, contributing to cancer risk [76]. Immune cell infiltration contributes to creating an immunosuppressive microenvironment, inhibiting antitumor immune responses. Obesity may also alter adipose stem cell function, disrupt tissue homeostasis, and induce hypoxia, leading to a proinflammatory state, increased angiogenesis, and oxidative stress that damages DNA [2, 38, 52, 77]. Similar to premenopausal women, emerging evidence on obesity altering the breast tissue microbiome and how it can influence MAT function, immune responses, and BC risk by promoting inflammation or altering hormone metabolism is applicable for postmenopausal women as well [70].

While hormone receptor–positive cancers are the most common among postmenopausal women, obesity also increases the risk of more aggressive subtypes like triple-negative BC. The chronic inflammation and immune suppression associated with MAT dysfunction is suggested to be the key drivers for the development of these aggressive subtypes.

The combined effects of chronic inflammation, hormonal imbalance, metabolic reprogramming, ECM remodeling, altered cell signaling and gene expression, and age-associated changes in postmenopausal obese women create a tumor-promoting feedback loop. This feedback loop involves inflammation-enhancing aromatase activity in MAT, leading to increased local estrogen production; estrogen promoting the proliferation of cancer cells and recruiting immune cells that sustain chronic inflammation; and metabolic alterations providing essential nutrients, adipokine imbalance, and immune cell recruitment that together fuel cancer growth. Thus, the shift from ovarian to MAT-derived estrogen makes MAT dysfunction central to BC risk, especially for ER+ subtypes [12]. Therefore, addressing MAT dysfunction through therapeutic strategies and lifestyle interventions could reduce postmenopausal BC risk and improve outcomes.

## 7. Translating mammary adipose tissue dysfunction mechanisms into prevention and therapeutic strategies

Understanding the role of MAT dysfunction in BC risk has significant therapeutic implications, particularly for postmenopausal women. Addressing the complex interplay between obesity, inflammation, hormonal imbalance, and metabolic reprogramming in MAT can open new avenues for BC prevention and treatment. The choice of therapeutic intervention is based on the grade, stage, and BC molecular subtype to ensure a precise, personalized, safe, and efficient therapy. The first line of treatment for most solid tumors, like in BCs, continues to be surgical removal—either lumpectomy or mastectomy. This is usually paired with radiotherapy to eliminate residual cancer cells and reduce the risk of recurrence, or in many cases, systemic therapies such as neoadjuvant or adjuvant chemotherapy to shrink tumors, prevent metastasis, and improve long-term outcomes [78]. However, these approaches are not always sufficient for all molecular subtypes, leading to a need for personalized, targeted therapies. In response to the growing need, new treatments addressing hormonal, immunological, and metabolic drivers of tumor growth have been developed for improved outcomes. As a result, based on the accumulating evidence that highlights the role of obesity-induced MAT dysfunction, interventions to restore homeostasis within the MAT, like strategies to correct hyperinsulinemia, chronic inflammation, and aromatase-driven estrogen production are potential therapeutic options beyond standard therapy.

Current therapies targeting the TME offer promise for improving BC outcomes and prevention. Aromatase inhibitors, already used for hormone receptor–positive BC, may help prevent BC in high-risk women and postmenopausal women where MAT is the predominant source of estrogen. Targeting estrogen signaling pathways to prevent the growth of hormone-driven BCs using ER modulators (selective ER modulators) and degraders (selective ER degraders) has also shown potential [78, 79] as they can target hormone-driven proliferation, directly counteracting the MAT-derived estrogen surplus.

Addressing obesity-related inflammation and metabolic dysfunction is another promising avenue. Anti-inflammatory drugs like NSAIDs and COX-2 inhibitors reduce inflammation and CLS formation in the MAT, with regular use linked to lower BC risk in postmenopausal women [78, 80]. Emerging approaches aim to modulate ECM remodeling, immune cell dynamics, and epigenetic regulation. One such approach is using collagenase inhibitors to restrict tumor invasion. Hyperinsulinemia and IR fuel tumor proliferation through activation of the PI3K/AKT and MAPK pathways. Therefore insulin-sensitizing agents like metformin and glucagon-like peptide-1 receptor agonists are being explored to lower circulating insulin levels and correct downstream signaling. Leptin levels are elevated in obesity, which contributes to JAK/STAT3, PI3K/AKT, and MAPK/ERK pathway activation, promoting cancer cell proliferation, migration, invasion, and stemness. Therefore, targeting leptin offers a novel, pathway-specific intervention with potential to disrupt tumor-promoting metabolic crosstalk, and therefore different methods to achieve this, like leptin antagonists and leptin peptide receptor antagonists, are being explored [81, 82]. Immunotherapies, including checkpoint inhibitors (anti-PD-1, PD-L1, CTLA-4), have demonstrated efficacy in certain BC types, especially triple-negative, while targeting cytokines and metabolic pathways can help restore immune balance and reduce inflammation in obesity-associated BC [83–85]. Additionally, the matrix metalloproteinases secreted by the CAAs can be therapeutically targeted by inhibitors to limit cancer cell invasion and metastasis, and these are actively being explored in clinical trials [86].

Lifestyle changes like weight loss, healthy diet, and regular exercise play a key role in reducing BC risk, especially in postmenopausal women. Weight loss lowers estrogen, inflammation, and IR, while physical activity improves adipokine balance and reduces tumor growth, recurrence, and mortality. Physically active women have an estimated 20% lower risk of developing BC compared with sedentary women [87]. The Women's Health Initiative Observational Study reported that postmenopausal women who lost 5% or more of their body weight had a 12% decrease in BC risk [13], with more significant reductions noted in hormone receptor–positive cases (∼15%–25%). Even modest weight loss reduces proinflammatory structures in mammary fat. Diets rich in plants and healthy fats—especially the Mediterranean diet—are linked to lower BC risk and improved outcomes, with studies showing up to 62% reduction in invasive BC among women adhering to a Mediterranean diet, supplemented with extra-virgin olive oil [88]. Fasting-mimicking diets also enhance treatment response by targeting cancer-related metabolic pathways [89].

Taken together, therapeutic strategies that address the drivers of MAT dysfunction, primarily hormonal imbalances, chronic inflammation, metabolic syndrome, and immune dysregulation, offer the potential for more effective BC prevention and management, especially in obese postmenopausal women. Combining lifestyle interventions with these therapeutic options offers an effective strategy for BC prevention and management in obese women.

## 8. Conclusion

Obesity-driven dysfunction of MAT emerges as a central mechanism in influencing the BC risk. Key mechanisms such as aromatase overexpression, immune cell infiltration, ECM remodeling, and epigenetic changes contribute to creating a tumor-supportive microenvironment in MAT. These changes establish a feedback loop that sustains inflammation, hormone dysregulation, and metabolic dysfunction, increasing cancer risk. This is further amplified by bidirectional crosstalk between adipocytes and cancer cells, which fosters immune evasion, metabolic reprogramming, and ECM remodeling.

From a clinical standpoint, the mechanistic insights discussed in this review will aid in better understanding, and thereby, the development of novel strategies for effective treatment of BC in this population. Understanding the distinct mechanisms of MAT dysfunction, especially in cases of pre- and postmenopausal women, has important implications for BC prevention and treatment. Given this, future research should explore further the possibility of correcting MAT dysfunction, including developing selective aromatase inhibitors, restoring adipokine balance, and modulating metabolic pathways to counteract obesity-driven BC progression. Immunotherapeutic approaches, including immune checkpoint inhibitors and metabolic modulators, targeting proinflammatory cytokines, and exploring oncolytic viruses and MMP inhibitors, offer promising avenues for improving treatment efficacy and reducing BC risk. Additionally, combining pharmaceutical approaches with lifestyle therapies like exercise and nutritional changes could enhance patient outcomes. A multidisciplinary strategy combining immunology, metabolomics, oncology, and endocrinology will be necessary to translate these discoveries into clinical practice. Risk assessment should also move beyond traditional BMI measurements, which fail to capture the extent of obesity’s’ impact on BC. More accurate body composition measurements, such as dual-energy X-ray absorptiometry or bioelectrical impedance analysis, alongside genetic and metabolic profiling, could enable more precise and personalized risk stratification [90].

Ultimately, addressing MAT dysfunction as a modifiable factor in BC development offers a tangible path to reducing obesity-associated cancer burden. By integrating targeted therapies, precision medicine, and public health initiatives, we can take meaningful strides toward reducing BC incidence and improving outcomes for women worldwide.

## Data Availability

No new data were generated or analysed in support of this review.
